# Modification of Soft Wheat Protein for Improving Cake Quality by Superheated Steam Treatment of Wheat Grain

**DOI:** 10.3390/foods12162992

**Published:** 2023-08-08

**Authors:** Yuanxiao Liu, Mengmeng Li, Erqi Guan, Yuanfang Liu, Ke Bian, Yingquan Zhang

**Affiliations:** 1 College of Food Science and Engineering, Henan University of Technology, Zhengzhou 450001, China; liuyuanxiao1991@163.com (Y.L.); limeng112578@126.com (M.L.); guanerqi@163.com (E.G.); 2 College of Chemistry and Chemical Engineering, Zhengzhou Normal University, Zhengzhou 450001, China; liuyuanfang328@zznu.edu.cn; 3 Institute of Food Science and Technology, Chinese Academy of Agricultural Sciences/Key Laboratory of Agro-Products Processing, Ministry of Agriculture and Rural Affair, Beijing 100193, China; zhangyingquan@caas.cn

**Keywords:** superheated steam (SS), wheat protein structure, foaming properties, emulsifying properties, specific volume of cakes, texture of cakes

## Abstract

Many varieties of soft wheat in China cannot fully satisfy the requirements of making high-quality cakes due to their undesirable protein properties, which leads to shortages of high-quality soft wheat flour. Therefore, a modification of soft wheat protein is essential for improving the quality of soft wheat and thus improving cake quality. In order to modify the protein properties of soft wheat used for cake production, superheated steam (SS) was used to treat soft wheat grains at 165 °C and 190 °C for 1, 2, 3, 4, and 5 min, respectively, followed by the milling of wheat grains to obtain refined wheat flour. The properties of proteins and cakes were analyzed using refined wheat flour as materials. First, changes in the structures of wheat proteins were analyzed by determining the solubility, molecular weight distribution and secondary structure of proteins in wheat flour. Secondly, changes in the functional properties of proteins were analyzed by determining the foaming properties and emulsifying properties of proteins in wheat flour. Finally, the specific volume and texture of cakes with wheat flour milled from SS-treated wheat were analyzed. At the initial stage of SS treatment, some of the gliadin and glutenin aggregated, and the gluten macro-polymer (GMP) contents increased. This allowed a more stable gluten network to form during dough kneading, leading to an improvement in dough elasticity. In addition, a short time period (1–3 min) of SS treatment improved the emulsifying properties and foaming ability of wheat protein, which helped to improve the specific volume and texture of cakes. Increasing the SS temperature from 165 °C to 190 °C reduced the optimal treatment time needed to improve cake quality from 3 min to 1 min. SS treatment for longer time (>3 min) periods led to severe protein aggregation and a decrease in the foaming ability and emulsifying properties of protein, which led to a deterioration in the cake quality. Thus, SS treatment at 165 °C for 1–3 min and 190 °C for 1 min could be a suitable method of improving the physicochemical properties of soft wheat used to make cakes with high specific volumes and good texture.

## 1. Introduction

Cakes are a widely consumed wheat-based food and the output of cakes has rapidly increased in recent years. High-quality cakes usually have high specific volumes, soft tastes, and a smooth, uniform, and fine texture. Wheat flour quality attributes that are closely related to cake quality include wet gluten content, dough stability, ash content, and particle size, among which dough stability is the key attribute. Wheat flour with a high dough stability limits the extension of cake batter during baking, thereby hindering the formation of cakes with high specific volumes. Therefore, soft wheat flour is usually used as a main material for cakes. However, many of the soft wheat varieties in China have unstable physicochemical qualities, which leads to a shortage of high-quality soft flour for producing cakes with good qualities. Cake quality is strongly associated with the properties of wheat protein as it is a main component in soft wheat flour. Wheat protein consists of albumin, globulin, gliadin, SDS-soluble glutenin, and SDS-insoluble glutenin. The solubilities, molecular weights (MWs), molecular structures, and functional properties of proteins in wheat flour are all closely related to cake quality [1,2]. Heat treatment of wheat or wheat flour has been widely used to modify soft wheat in order to improve cake quality [3,4,5]. These modifications cause significant changes in the protein structures, such as glutenin aggregation, protein denaturation, sulfydryl–disulfide bond exchange reactions, and an increase in the α-helices percentage, which are attributed to be one of the main reasons for improvement in the cake quality. 

Superheated steam (SS) is a modern development in heat treatment methods used to process wheat [6,7]. All wheat proteins are heat-sensitive, but their resistance to heat varies significantly. Albumins and globulins are more thermostable than gliadins and glutenins, and α-gliadins are more thermostable than β-gliadins and γ-gliadins [8,9]. Previous studies have suggested that modest SS treatment of wheat flour can increase the gluten strength, whereas excessive SS treatment may considerably damage wheat gluten [10,11]. The functional properties of wheat protein may significantly change after the SS treatment of wheat. Cake batter was prepared using wheat flour, butter, egg, and other accessory materials, among which wheat proteins contribute to the formation of networks. In addition, the foaming and emulsifying properties of wheat and egg proteins contribute to the formation of cakes with specific volumes and textures during baking. Therefore, changes in the functional properties of wheat proteins may modify the specific volume and texture of cakes. Changes in the functional properties of protein and cake qualities may be strongly associated with the solubilities, components, disulfide bonds, MWs, and secondary structures of wheat proteins. Compared with SS treatment of wheat flour, SS treatment of wheat grain may have significantly different effects on the structures of protein because of the different heat transfer pathways and velocities. Although previous studies have proved that SS treatment of wheat flour and wheat grain at specific conditions can improve cake quality, the relationships between the changes in protein properties and cake qualities following the SS treatment of wheat grain are still unclear [6,12]. 

In our previous research, wheat grains were treated with SS at 115, 140, 165, 190, and 215 °C for 1, 3, 5, 7, and 9 min, respectively. We found that the use of appropriate times and temperatures for SS treatments (SS treatments at 165 °C and 190 °C for 1 and 3 min and at 215 °C for 1 min) of wheat grain can improve the specific volume and texture of cakes, whereas an excessive treatment time may damage cake quality [6]. Therefore, we have set new conditions of SS treatment for this research. In this research, soft wheat grains were treated with SS at 165 °C and 190 °C for between 1 and 5 min. The SS-treated wheat flour was then milled to analyze changes in the solubilities, compositions, MWs, structures, and functional properties of the wheat protein. This wheat flour was then used to make cakes that were then analyzed for their specific volumes and textures. This study aims to demonstrate the effects of SS treatment on the structures and physicochemical properties of wheat protein and their relationships with cake quality.

## 2. Materials and Methods

### 2.1. Materials

Soft red winter wheat grains with a moisture content of 11.2%, a protein content of 10.2%, a damaged starch content of 4.1%, a falling number of 245 s, a wet gluten content of 24.9% and a dough stability time of 1.5 min were purchased from Runyang Seed Co., Ltd. (Yangzhou, China). SS treatments of wheat grain were conducted after the harvesting of wheat for 1.5 years.

### 2.2. SS Treatment and Wheat Milling

Wheat grains were standardized to a moisture of 13%. After 24 h of stabilization, wheat grains were treated using an SS processing instrument (DLSH-50, Dingli Co. Ltd., Zhengzhou, China) at 165 °C and 190 °C for 1 min, 2 min, 3 min, 4 min, and 5 min, respectively. SS-treated wheat grains were standardized to the moisture content of 15% and milled using experimental flour mill (MLU-202, Uzwil, Switzerland) to obtain refined wheat flour with a flour extraction rate of 65% for further use.

### 2.3. Solubilities of Wheat Protein

Wheat protein was extracted from wheat flour according to a modified Osborne method [13]. Deionized water (10 mL) was added to wheat flour (1.00 g) and mixed uniformly in a centrifuge tube. The suspension in the centrifuge tube was stirred using a magnetic stirrer at 50 °C for 30 min, followed by spinning in the centrifuge at 4000 r/min for 20 min. The supernatant was diluted to 50 mL (Solution A) in order to determine its albumin contents. 

We added 10 mL of sodium chloride (10%, *w*/*v*) into the above sedimentation. Globulin was extracted according to the Osborne method and diluted to 50 mL (Solution B) for the determination of globulin contents. 

After globulin extraction, the sedimentation was mixed with 10 mL of ethanol (70%, *v*/*v*). The suspension was stirred at 50 °C for 5 min, followed by stirring at room temperature for 30 min. This suspension was centrifuged at 4000 r/min for 20 min and the supernatant was diluted to 50 mL (Solution C) for the determination of gliadin contents.

After gliadin extraction, the sedimentation was mixed with 10 mL of sodium hydroxide (0.2%, *w*/*v*). Glutelin was extracted according to the specifications of the Osborne method and diluted to 50 mL (Solution D) for the determination of glutelin contents.

Solutions A, B, C, D, and the final sedimentation were used for the determination of albumin, globulin, gliadin, glutenin, and GMP contents, respectively. Protein content determination was conducted according to the Kjeldahl method using an automatic Kjeldahl apparatus (KJELTEC 8400, FOSS Co. Ltd., Copenhagen, Denmark) and calculated with a protein conversion coefficient of 5.70.

### 2.4. Size Exclusion High-Performance Liquid Chromatography (SE-HPLC)

Size exclusion high-performance liquid chromatography (SE-HPLC) was used to analyze the protein compositions of wheat flour and the determination was conducted according to the method proposed by Lagrain et al. [14] with a slight modification. A total of 0.05 g of wheat flour was mixed with 5 mL of solvent A (containing 0.05 mol/L phosphate-buffered solution (PBS) with a pH of 6.9 and 2% (*w*/*v*) SDS) and vibrated at 2500 r/min for 60 min, followed by centrifugation at 4000 r/min for 20 min. A total of 1.5 mL of the supernatant was centrifuged at 8000 r/min for 10 min before filtration through a microfiltration membrane with a pore size of 0.45 μm. The solution after filtration was transferred into a sample bottle for HPLC analysis. Solvent B (solvent A containing 2 mol/L urea and 1% (*w*/*v*) dithiothreitol (DTT)) was used for the extraction of wheat protein after the reduction of GMP. HPLC analysis was performed on an HPLC system (e2695, Waters, Milford, CT, USA) using an SEC column (Biosep s4000, Phenomenex, Torrance, CA, USA). The elution solvent was water/acetonitrile (50/50, *v*/*v*), containing 0.1% trifluoroacetic acid (TFA). A total of 60 μL of each prepared sample was injected and analyzed at a flow rate of 0.5 mL/min from 0 to 30 min and UV absorbance was determined at the wavelength of 214 nm. 

### 2.5. Reversed-Phase High-Performance Liquid Chromatography (RP-HPLC)

Reversed-phase high-performance liquid chromatography (RP-HPLC) was used to further analyze the protein compositions of wheat flour according method proposed by Wiser et al. [15] with a slight modification. 

Extraction of albumin and globulin. A total of 0.25 g wheat flour was mixed with 2.5 mL solution A (pH = 7.6), which contains 0.4 mol/L of sodium chloride and 0.067 mol/L of sodium hydrogen phosphate. After being stirred with an electric stirrer at 2500 r/min for 15 min, the sample was centrifuged at 6000× *g* for 15 min. The supernatant was transferred and another 2.5 mL of solution A was added into the centrifuge tube for a second extraction. The supernatants were consolidated after the two extractions and diluted to 5 mL for the determination of albumin and globulin.

Extraction of gliadin. The sedimentation after the extraction of protein sample A was extracted with 1.25 mL of solution B (70% (*v*/*v*) ethanol) twice according to the Wiser method. The supernatants were consolidated after thetwo extractions and diluted to 5 mL (protein sample B) for the determination of gliadin.

Extraction of glutenin. The sedimentation after the extraction of albumin, globulin, and gliadin was mixed with 2.5 mL of solution C, which contained 50% (*v*/*v*) of propyl alcohol, 2 mol/L of urea, 0.05 mol/L of tris-HCl-buffered solution with pH of 7.5 and 1% (*w*/*v*) of DTT. The mixed solution was stirred with an electric stirrer at 2500 r/min for 15 min before being heated in a water bath to 60 °C within a nitrogen environment for 20 min. The supernatant was collected and, after two extractions, the supernatants were merged and diluted to 5 mL for further use.

The mobile phase included A (water containing 0.1% (*v*/*v*) trifluoroacetic acid) and B (acetonitrile containing 0.1% (*v*/*v*) trifluoroacetic acid). For albumin and globulin, the elution procedure began with 80% A and ended with 40% A after 30 min. For gliadin and glutenin, the elution procedure began with 72% A and ended with 44% A after 30 min. The UV absorbance was monitored at the wavelength of 220 nm.

### 2.6. SDS-PAGE

A sample solution containing 0.05 mol/L tris-hydrochloric acid-buffered solution with pH of 6.8, 10% (*w*/*v*) SDS, 10% (*v*/*v*) glycerin, and 0.1% (*w*/*v*) bromophenol blue was prepared in advance of the experiment for use in sodium dodecyl sulfate–polyacrylamide gel electrophoresis (SDS-PAGE). A total of 50 mg wheat flour was accurately weighed and mixed with 1.0 mL of sample solution; the resultant mixture was vibrated at 2500 r/min for 10 min. Sixty minutes later, the samples were heated in a boiling water bath for 10 min before undergoing centrifugation at 18,500× *g* for 10 min.

A cast of 12% separation gel with a pH of 8.8 and 5% spacer gel with a pH of 6.8 was allowed to form for 60 min. Then, the sample was injected with an 8 μL protein marker (Thermo Scientific Co. Ltd., Waltham, MA, USA) containing proteins of 180, 130, 100, 70, 55, 40, 35, 25, 15, and 10 kDa and 20 μL sample solutions into sample cells. A voltage of 80 V with an electric current of 25 mA were used in the concentration stage and a voltage of 100 V with an electric current of 25 mA were used in the separation stage. After separation, the gel was dyed using Coomassie brilliant blue for 2 h, followed by decolorization for 2 h. The decolored gel was scanned using a gel imager (WD-9413C, Liuyi Biotech. Co. ltd., Beijing, China).

### 2.7. Secondary Structure

The secondary structure of the wheat protein was determined using Fourier transform infrared spectroscopy (FT-IR) via the method proposed by Misra et al. [16] with a slight modification. In total, 2 mg wheat flour and 198 mg potassium bromide were mixed in an agate mortar. The mixture was compacted into a 2 mm-thick slice. FT-IR was used to analyze samples at the wavenumber range of 4000–400 cm^−1^ with a resolution ratio of 4 cm^−1^ and 200 scans were conducted for each sample.

### 2.8. Foaming Properties

Foaming properties were determined according to the method proposed by Don et al. [17] with a slight modification. A 2.0 g sample (wheat flour or gluten extracted from the homologous wheat flour) was uniformly mixed with 100 mL of PBS with the concentration of 0.01 mol/L and pH of 7.0. In total, 20 mL of the mixed solution was homogenized at 10,000 r/min for 2 min; the volume (*V*_1_) of the solution after homogenization was recorded. The volume (*V*_2_) of the solution after resting for 30 min was recorded. Foaming ability (FA) and foaming stability (FS) were calculated according to the following formulae:

(1) $$\mathrm{FA}=\frac{V_{1}-20}{20}\times 100\%$$

(2) $$\mathrm{FS}=\frac{V_{2}}{V_{1}}\times 100\%$$

### 2.9. Emulsifying Properties

The emulsifying properties of wheat protein were determined according to the method proposed by Pearce [18] with a slight modification. A 1 g sample (wheat flour or gluten extracted from the homologous wheat flour) was uniformly mixed with a 100 mL phosphate buffer with the concentration of 0.2 mol/L and pH of 7.0. A volume of 35 mL of this solution was mixed with 10 mL of peanut oil before homogenization at 10,000 r/min for 1 min. A volume of 50 μL of the homogenized solution was mixed with 5 mL of 0.1% (*w*/*v*) SDS solution after 0 and 10 min, respectively, before the absorbance at 500 nm (A_500_) was measured. Emulsifying activity index (EAI) and emulsion stability (ES) were calculated as follows:

EAI = (4.606 × C × A_500_ × N × 10^−4^)/(ΦCL) (3)

ES = (*A*_0_ × ΔT)/(*A*_10_ − *A*_0_) (4)

where N is the dilution ratio; C is protein concentration (g/mL); Φ is volume fraction of oil phase; L is cuvette light diameter; *A*_0_ is absorbance at 0 min; *A*_10_ is absorbance at 10 min; and ΔT is the time difference value.

### 2.10. Cake Making

Cakes were made according to the method proposed by Liu et al. [6] with a slight modification. The formulation of cakes included 100 g of wheat flour, 80 g of butter, 50 g of caster sugar, 55 g of egg albumen, 30 g of egg yolk, 15 g of water, 3 g of milk powder, 2 g of baking powder, and 0.3 g of salt. First, the caster sugar, egg albumen, egg yolk, water, and salt were put into a bowl and stirred for 3 min. Second, the wheat flour, milk powder, and baking powder were mixed with the aforementioned ingredients while being continuously stirred for 5 min. Then, the butter which had been melted using a water bath in advance was added into the bowl and stirred rapidly for 3 min. The prepared batter was placed in a mold with a length of 100 mm and a width of 50 mm and baked at 180 °C for 20 min. 

### 2.11. Specific Volumes of Cakes

Cake volume (mL) was determined using the rapeseed replacement method and cake weight (g) was measured using an analytical balance (GL224I-1SCN, Sartorius, Göttingen, Germany). Specific volumes (mL/g) were calculated as the ratio of cake volume to cake weight [6].

### 2.12. Texture of Cakes

The texture of cakes was determined using a TA-XT 2i texture analyzer (Stable Microsystems Co. Ltd., Surrey, UK) according to the method proposed by Liu et al. [6] with minor modifications. The cake crumbs were cut into cubes with edge lengths of 25 mm. The texture of cakes was determined using the following conditions: a pre-test speed, testing speed and post-test speed of 1 mm/s; a trigger force of 10 g; a compression depth of 50%; and a 30 s time delay between the two compressions. The hardness (g), springiness, cohesiveness, chewiness (N), and resilience of cakes were recorded and 10–15 duplicates were conducted for each sample.

### 2.13. Statistical Analysis

For each experiment, at least three parallel tests were conducted. The mean and standard deviation (SD) of parallel values were calculated and the results were displayed as mean ± SD. One-way analysis of variance and a Duncan multiple comparison were conducted using SPSS 26.0 (SPSS Institute, Chicaco, IL, USA). Results with *p* values of less than 0.05 were considered significantly different. All graphs were drawn using Origin 2018 (Origin Lab Corporation, Northampton, MA, USA).

## 3. Results and Discussion

### 3.1. Solubility of Wheat Protein

Wheat proteins can be divided into albumin, globulin, gliadin and glutenin according to their solubility. The protein structures, such as sulfydryl/disulfide bond ratio, secondary structure types and ratios, and molecular weights, can significantly change during the SS treatment of wheat, leading to a change in protein solubility and therefore a change in the amounts of each protein type [19,20,21,22]. The changes in the contents of albumin, globulin, gliadin, and glutenin in wheat flour following SS treatment of wheat are shown in Table 1.

During SS treatment, heat transfers into wheat grain, which may cause conformational and structural changes in wheat protein. SS temperature, time, and SS condensation are all related to these changes. The condensation time of SS at 190 °C is shorter than that at 165 °C, which may lower the time necessary for the changes to take place in protein structures. An SS treatment of wheat at 165 °C led to a decrease in the albumin content and an increase in the globulin content and the GMP content at specific conditions. As the treatment time was extended, the albumin content continued to decrease, while the gliadin and glutenin contents increased from 0 to 2 min of SS treatment and decreased from 2 to 4 min of SS treatment. Similar variations were observed in the trends for the albumin, globulin, and gliadin contents at 190 °C of SS treatment. The glutenin content increased from 0 to 2 min and the GMP content increased from 0 to 4 min at 190 °C of SS treatment. The aforementioned results showed that part of the albumins may be transformed into globulins, while another part of the albumins may be transformed into proteins with higher molecular weights. This transformation occurs because of the reduction in electrostatic repulsive force and the heightening of hydrophobic interactions between albumin molecules, both of which result from the albumin aggregation during SS treatment. The increase in globulin contents may help to improve the emulsifying properties of wheat protein. The amounts of each protein type in wheat flour significantly changed after SS treatment of wheat, which may have been mainly because of the aggregation of albumin, gliadin and monomeric glutenin [23,24]. The increase in the amounts of gliadin, glutenin, and GMP at 1–3 min of SS treatment helped to improve to the extensibility and elasticity of cake dough, thus improving the specific volume and texture of cakes.

### 3.2. SE−HPLC

**Figure 1:**
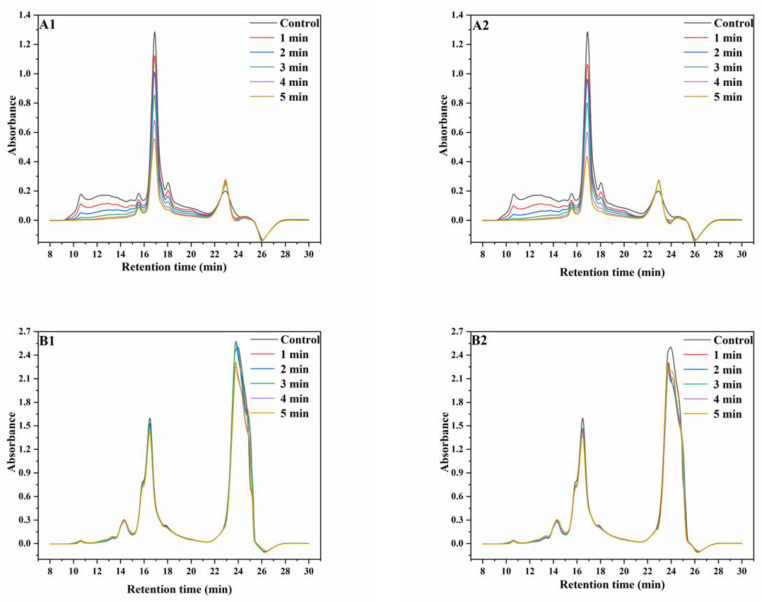
SE−HPLC graphs of wheat protein. ((**A1**), non-reducing SE−HPLC graphs of protein extracted from wheat treated with superheated steam at 165 °C; (**A2**), non-reducing SE-HPLC graphs of protein extracted from wheat treated with superheated steam at 190 °C; (**B1**), reducing SE−HPLC graphs of protein extracted from wheat treated with superheated steam at 165 °C; (**B2**), reducing SE−HPLC graphs of protein extracted from wheat treated with superheated steam at 190 °C).

Wheat proteins have a wide range of molecular weight distribution (MWD). During the SS treatment of wheat, the aggregation of monomeric proteins and the changes in disulfide bond contents can cause significant changes in the MWD of wheat proteins [25]. SE-HPLC is appropriate for use in the analysis of proteins with wide ranges of MWDs [26] and the results of wheat proteins before and after SS treatment are shown in Figure 1.

Under non-reducing conditions at 165 °C, the intensities of the peaks at the retention times (RT) of 10–11 min, 14–16 min, an 16–18 min significantly decreased with increasing SS treatment time, whereas a slight increase was observed in peak intensities at the RT of 21–24 min. The trend of the changes in peak intensity with increasing SS treatment time at 190 °C were similar to that at 165 °C. Under reducing conditions at 165 °C, the intensities of peaks at the RT of 15–17 min decreased with increasing SS treatment time, whereas the intensities of peaks at the RT of 22–25 min initially increased and then decreased. At 190 °C, the intensities of both peaks decreased with increasing SS treatment time. Meanwhile, the chromatograms showed that the contents of proteins with high molecular weights (RT, 10–11 min) decreased more significantly than those of proteins with low molecular weights (RT, 16–18 min) due to the differences in heat stability among different protein types [8,9]. The aforementioned results showed that the amounts of proteins with most MWs significantly decreased, although the MWD curves were similar. This may be because of the aggregation of monomeric proteins, which significantly increased the amounts of high-molecular proteins that are insoluble in SDS, therefore leading to the significant decrease in SDS extractability of wheat proteins [24]. This result is in accordance with the result of the increasing content of GMP after SS treatment, as described in Section 3.1. Additionally, most but not all of the aggregated proteins depolymerized after the reduction reaction with DTT, which indicated that proteins aggregated mainly through disulfide bonding [27]. 

### 3.3. RP−HPLC

**Figure 2:**
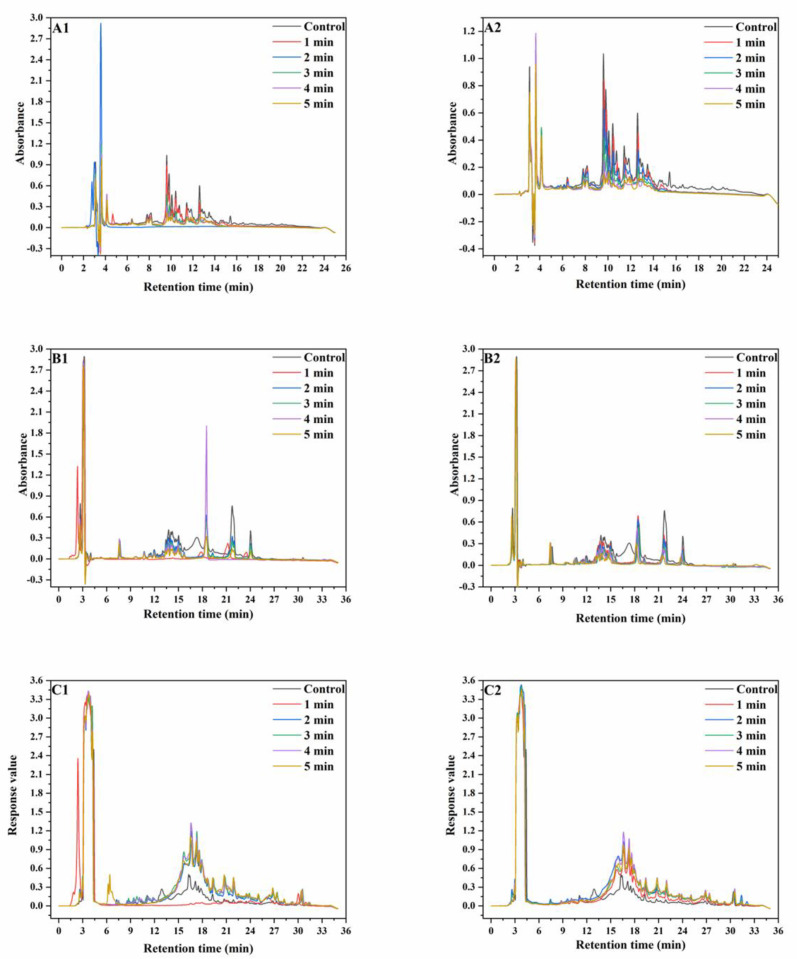
RP−HPLC graphs of wheat protein. ((**A**), RP−HPLC graphs of wheat albumin and globulin; (**B**), RP−HPLC graphs of wheat gliadin; (**C**), RP−HPLC graphs of wheat glutenin; (**A1**–**C1**), RP−HPLC graphs extracted from wheat treated at 165 °C; (**A2**–**C2**), RP-HPLC graphs extracted from wheat treated at 190 °C).

RP−HPLC is more efficient than SE−HPLC at separating proteins, but can only be used to separate proteins with lower MWs (≤100 kDa) [28]. In this paper, RP−HPLC was used to analyze albumins, globulins, gliadins, and glutenins reduced by DTT. The results are shown in Figure 2. 

The peak intensities of albumins and globulins decreased after the SS treatment and continued to decrease with increasing SS treatment time. The variation in peak intensities at 190 °C was more significant than that at 165 °C. In the chromatograms of gliadins, the peaks at the RT of 6–9 min, 10–18 min and 18–25 min represent ω-gliadin, α-gliadin, and γ-gliadin, respectively. At both 165 °C and 190 °C, there was no observable change in the peak intensity of ω-gliadin. This was because ω-gliadins were short of cysteine and methionine and were difficult to aggregate through disulfide bonding. With increasing SS treatment time, the peak intensities at 22 min and 24 min significantly decreased, whereas the peak intensity at 19 min initially increased and then decreased. SS treatment led to the disappearance of the peak at 17 min. This was because α-gliadin and γ-gliadin were rich in sulfur amino acids and might aggregate through disulfide bonding under SS treatment. In the chromatograms of reduced glutenin, peak intensities significantly increased after SS treatment, which might be because of the reduction of the disulfide bonds in the aggregated un-glutenin proteins, thus resulting in an increase in monomeric glutenin content. The aforementioned results showed that after SS treatment of wheat, partial albumin, globulin, and gliadin polymerized, while partial gliadins depolymerized [29,30]. As a whole, the monomeric protein content in wheat flour significantly decreased after SS treatment. 

### 3.4. SDS-PAGE

**Figure 3:**
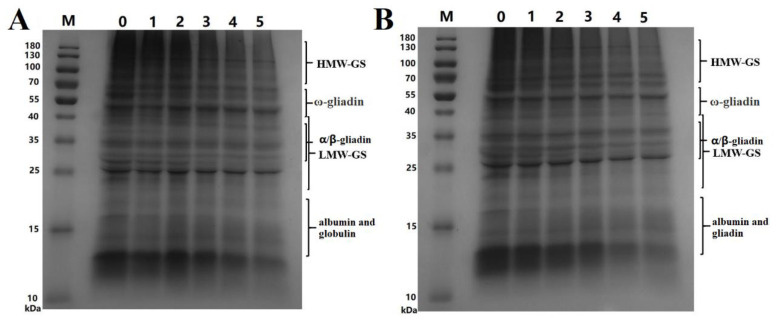
SDS-PAGE electrophoretograms of wheat protein. ((**A**), SDS-PAGE graphs of protein extracted from wheat treated at 165 °C; M, marker; 0, control sample; 1, 2, 3, 4, and 5 represent the treatment time of 1–5 min, respectively. (**B**), SDS-PAGE graphs of protein extracted from wheat treated at 190 °C; M, marker; 0, control sample; 1, 2, 3, 4 and 5 represent the treatment time of 1–5 min, respectively. HMW-GS, high-molecular-weight glutenin subunit; LMW-GS, low-molecular-weight glutenin subunit).

SDS-PAGE was used to further analyze the changes in MWDs of wheat proteins after SS treatment of wheat and the results are shown in Figure 3.

The changes in the contents of proteins with different MWs can be inferred from the electropherograms. According to the contents of Figure 3, we inferred that albumin and globulin contents in wheat flour significantly decreased after SS treatment of wheat. This decrease was mainly attributed to the decrease in the albumin content, which had been described in Section 3.1. The gliadin and glutenin contents significantly decreased when treatment time exceeded 3 min. However, the decrease in-gliadin was not very obvious, which was because that ω-gliadin was short of sulfur amino acids. The albumin content decreased significantly faster than the levels of globulin, gliadin, and glutenin. All proteins decreased faster at 190 °C than at 165 °C. These results showed that protein aggregation occurred during the SS treatment of wheat and that the extractability significantly decreased following the SS treatment. 

### 3.5. Secondary Structure

The secondary structures are closely related to the spatial structures of wheat proteins, thus affecting the physicochemical properties of wheat [31]. As a result, cake qualities can be associated with the secondary structures of wheat proteins. The results of changes in protein secondary structure after SS treatment are shown in Table 2.

Increasing SS treatment time at 165 °C caused the contents of α-helix, β-sheet, and random coil to initially increase and then decrease, while the contents of β-turn significantly decreased. The contents of α-helix, β-sheet, and random coil reached maximums after treatment times of 3 min, 2 min, and 2 min, respectively. At 190 °C, similar trends were observed with increasing treatment time, and the contents of α-helix, β-sheet, and random coil reached maximums after a treatment time of 2 min, 1 min, and 2 min, respectively. These results indicated that a short duration of SS treatment promoted the formation of α-helix and β-sheet structures, a process which was stabilized by hydrogen bonds. This might be associated with the increase in the free sulfhydryl content after the SS treatment. However, the α-helix and β-sheet structures formed during the SS treatment were not stable and were easily damaged after a long duration of SS treatment. Additionally, SS treatment led to conformational changes in wheat protein, thus increasing the random coil content at a short period of SS treatment. The increasing in the content of α-helix, β-sheet, and random coil might contribute to the improvement in the specific volume and texture of cakes.

### 3.6. Foaming Properties

During the production of cakes, the foaming properties (foamability (FA) and foaming stability (FS)) of cake batter are strongly correlated with the specific volumes of cakes [32]. Gluten networks and embedded starch granules constitute the basic structures of cakes. Gluten strength and starch viscosity determine the stability of cake structures. Foams formed from egg proteins and wheat proteins support the inner structure of cakes, thereby contributing to the volume, specific volume, and texture of cakes. The significant changes in the MWs and structure of wheat proteins observed with SS treatment could affect the foaming properties of wheat proteins and cake batter. The changes in the foaming properties of both wheat flour and wheat gluten after SS treatment were studied and the results are shown in Table 3.

With increasing SS treatment time at 165 °C, the FA of both wheat flour and wheat gluten initially increased and then decreased. No significant change was observed in the FS of wheat flour. The FS of wheat gluten showed no change from 0 to 2 min, but decreased significantly when the treatment time was extended from 2 to 5 min. The foamability and foaming stability of wheat flour reached maximums after 1 min and 3 min of treatment, respectively, while the foamability and foaming stability of wheat gluten reached maximums at 2 min and 1 min, respectively. At 190 °C, we observed similar trends in terms of foamabilities and foaming stabilities with increasing treatment time to those seen at treatment at 165 °C. The FA and FS of wheat flour reached maximums after 1 min treatment, whereas the FA and FS of wheat gluten reached maxims after 2 min and 1 min, respectively. A short period of SS treatment could improve the film-forming ability and hydrophobicity of proteins, thus helping proteins to rapidly absorb at the air–water interface during stirring [33]. As a result, the FA and FS of proteins were improved. Additionally, the improvement seen in FA and FS could also be related to the increase in the random coil of proteins. However, these properties were all damaged after a long period of SS treatment because of the severe denaturation of proteins. These results showed that SS treatment at suitable conditions could strengthen the FA of wheat proteins, which might help to improve the specific volumes, texture, and sensory qualities of cakes.

### 3.7. Emulsifying Properties

Lipids and wheat flour are the main materials of cakes. During the processing of cakes, wheat proteins play an essential role in reducing the agglomeration of oil drops and lowering the surface tensions of oil–water interfaces, thus enhancing the emulsifying stabilities of cake batter [34]. Therefore, the qualities of cakes are closely related to the emulsifying properties of wheat proteins. The changes in the emulsifying properties of wheat proteins caused by SS treatment were studied using wheat flour and wheat gluten as materials and the results are shown in Table 4.

For wheat flour at 165 °C, both the emulsifying activity index (EAI) and emulsion stability (ES) of wheat flour initially increased and then decreased with increasing treatment time. EAI and ES reached maximums following 2 min and 3 min of treatment, respectively. At 190 °C, the EAI of wheat flour initially increased and then decreased, whereas the ES of wheat flour initially decreased and then increased. EAI and ES reached maximums after 3 min and 5 min of treatment, respectively. Both the EAI and ES of wheat flour after SS treatment were higher than those of the control. For wheat gluten at 165 °C, EAI increased significantly with increasing treatment time, whereas ES initially increased and then decreased. EAI and ES reached maximums after 2 min and 4 min of treatment, respectively. At 190 °C, EAI initially increased and then decreased with increasing treatment time, whereas ES initially decreased and then increased. EAI and ES reached maximums after 3 min and 5 min of treatment, respectively. Both the EAI and ES of wheat gluten after SS treatment were higher than those of the control, which might be caused by the changes in the hydrophobicity, free sulfhydryl, disulfide bond, and secondary structure of wheat protein. These results showed that selected SS treatment conditions can significantly enhance the EAI and ES of wheat proteins, thus promoting the formation of stable emulsions after the stirring of wheat flour and butter. 

### 3.8. Specific Volumes of Cakes

**Figure 4:**
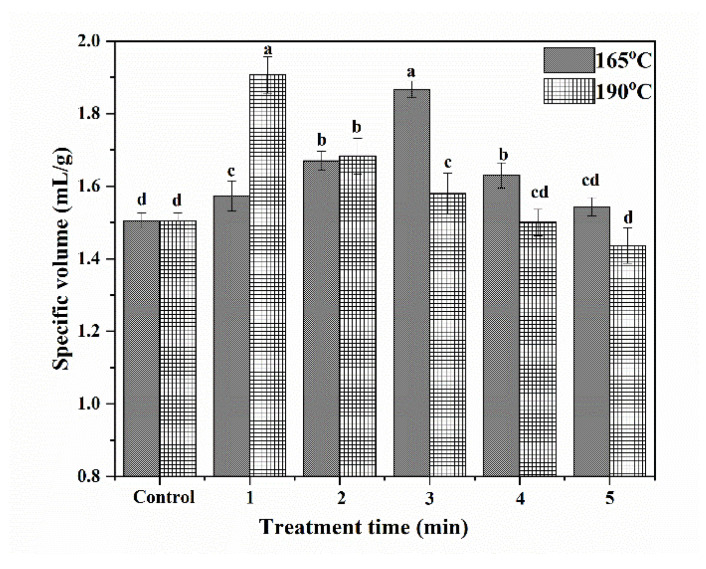
Specific volumes of cakes. (The bars were labeled with the letters a–d from the maximum to the minimum, such that bars with different letters on the same bars were significantly different (*p* < 0.05).

Specific volumes of cakes depend on the amount of air incorporated into cake batter and the gas retention capacity of cake batter, which are dependent on foaming properties of both egg proteins and wheat proteins, and cake batter viscosity [35,36,37]. The specific volumes of cakes made with wheat flour were studied both with and without SS treatment, and the results are shown in Figure 4. 

At both 165 °C and 190 °C, the specific volumes of cakes initially increased and then decreased with increasing SS treatment time. At 165 °C and 190 °C, the specific volumes of cakes reached maximums after 3 min and 1 min of treatment, respectively. These results showed that raising steam temperature can help to shorten the time needed to improv3 the specific volumes of cakes. A short period of SS treatment improved the contents of gliadin, glutenin, and GMP, thus helping to form a more stable gluten network during the preparation of cake batter. Additionally, increasing the FA of proteins could help to promote the formation of abundant bubbles, thus helping to improve the volume of cakes. As a result, the specific volume of cakes was significantly increased after the SS treatment of wheat. However, excessive SS treatment time can cause a reduction in the specific volumes. This could be because of the decrease in the content of gliadin, which reduced the extensibility of the cake batter network. Additionally, the severe denaturation of protein after a long period of SS treatment damaged the foaming properties of wheat protein, thus reducing the specific volume of cakes. 

### 3.9. Texture of Cakes

The texture of cakes at a specific volume determines the mouthfeel and taste of cakes. Cake texture is dependent on the number and diameter of air holes, networks, and fillers, which are the results of the physicochemical properties of protein and starch and their interactions. The effects of SS treatment of wheat on the texture of cakes were studied and the results are shown in Table 5.

At 165 °C, the hardness of cakes initially decreased and then increased with increasing treatment time. Meanwhile, springiness, cohesiveness, and resilience initially increased and then decreased with the extension of treatment time at 165 °C. However, chewiness did not change significantly with the extension of treatment time at 165 °C. Similar trends to those seen at 165 °C were observed at 190 °C. The conditions for improving the texture of the cakes were SS treatments at 165 and 190 °C for 1–3 min. The increase in the gliadin content after the SS treatment of wheat helped to improve the resilience of cakes since gliadin is closely related to the extensibility of cake networks. The increasing in the glutenin and GMP content after the SS treatment of wheat helped to improve the springiness since glutenin and GMP were closely related to the elasticity of cake network. Additionally, the decrease in the hardness of cakes occurred because of the increase in the specific volume of cakes, as explained in Section 3.8.

## 4. Conclusions

The SS treatments of soft wheat at 165 and 190 °C significantly affect the structures and properties of wheat proteins and therefore cake quality. At the initial stage of SS treatment, minimal amounts of heat are transferred into the endosperm as most of the heat is absorbed by the condensed water on the surface of wheat grains. This leads to more sulfhydryl groups being exposed, to some of the gliadins and glutenins aggregating and increases in GMP content. This causes more disulfide bonds to form during the kneading of dough compared to the control, thus improving dough elasticity. At both 165 and 190 °C, SS treatments for 1 to 3 min intensified the α-helix, β-sheet, random coil structures and hydrophobic properties of the wheat protein, allowing it to be easily absorbed at the oil–water interface and improving the emulsifying property and foaming ability of proteins. These changes in wheat protein properties improve the specific volume and texture of cakes. However, following longer treatment times (>3 min) at both 165 and 190 °C, large amounts of protein were aggregated and the amounts of SDS-unextractable protein significantly increased, thus leading to a decrease in the foaming ability and emulsifying property of wheat protein. All the factors led to a deterioration in cake quality. In conclusion, this work shows that SS treatments at 165 °C for 1 to 4 min and at 190 °C for 1 to 3 min can significantly improve the specific volume and texture of cakes. Based on the results of this study, SS treatments at 165 °C for 3 min and at 190 °C for 1 min can be chosen as the most appropriate methods for improving quality. However, further studies are still essential since we need to conduct technology optimization experiments in order to select the optimized conditions. In addition, the mechanisms of the changes in starch properties need to be studied in the future since they are closely related to the specific volume and texture of cakes. The industrialization of SS treatment needs to be taken into consideration in the future.

## Figures and Tables

**Table 1:** Effect of superheated steam treatment on the solubilities of wheat proteins.

Steam Temperature (°C)	Treatment Time (min)	Albumin (%)	Globulin (%)	Gliadin (%)	Glutenin (%)	GMP (%)
165	Control	4.24 ± 0.35 a	6.41 ± 0.43 b	34.21 ± 0.75 c	38.62 ± 1.35 bc	9.27 ± 0.45 d
	1	4.20 ± 0.11 a	6.15 ± 0.13 b	40.66 ± 0.56 b	40.75 ± 2.26 b	9.68 ± 0.45 d
	2	2.67 ± 0.04 b	7.50 ± 0.47 a	48.68 ± 1.11 a	45.32 ± 2.71 a	12.96 ± 0.19 c
	3	1.95 ± 0.02 c	7.80 ± 0.30 a	37.11 ± 2.23 bc	36.06 ± 1.65 c	17.32 ± 0.06 a
	4	1.60 ± 0.05 cd	7.79 ± 0.31 a	33.55 ± 0.93 c	35.43 ± 1.35 c	16.25 ± 0.35 b
	5	1.33 ± 0.01 d	8.21 ± 0.28 a	29.21 ± 2.23 d	48.30 ± 0.30 a	17.66 ± 0.42 a
190	Control	4.24 ± 0.35 a	6.41 ± 0.43 b	34.21 ± 0.75 b	38.62 ± 1.35 c	9.27 ± 0.45 d
	1	2.91 ± 0.26 b	6.35 ± 0.34 b	38.55 ± 1.30 a	38.62 ± 2.56 c	10.39 ± 0.16 d
	2	2.30 ± 0.07 c	6.88 ± 0.54 ab	41.32 ± 2.61 a	43.09 ± 1.66 ab	12.37 ± 0.06 c
	3	1.84 ± 0.14 c	7.45 ± 0.26 a	41.45 ± 2.42 a	42.66 ± 1.95 bc	15.68 ± 1.41 b
	4	1.36 ± 0.02 d	7.40 ± 0.13 a	31.19 ± 1.31 bc	46.49 ± 1.05 ab	17.39 ± 0.29 a
	5	1.28 ± 0.05 d	7.48 ± 0.21 a	28.55 ± 0.93 c	47.02 ± 0.91 a	14.96 ± 0.13 b

The data are given as the means of triplicate assays ± SD. The values were labeled with the letters a–d from the maximum to the minimum, such that values with different letters in the same column were significantly different (*p* < 0.05).

**Table 2:** Effect of superheated steam treatment on the secondary structure of wheat protein.

Steam Temperature (°C)	Treatment Time (min)	α-Helix (%)	β-Sheet (%)	β-Turn (%)	Random Coil (%)
165	Control	12.91 ± 0.12 f	36.85 ± 0.77 d	34.79 ± 0.76 a	11.11 ± 0.19 c
	1	13.16 ± 0.14 e	37.90 ± 0.43 c	32.51 ± 0.64 b	12.52 ± 0.32 b
	2	14.02 ± 0.10 c	41.20 ± 0.38 a	31.55 ± 0.46 bc	13.37 ± 0.23 a
	3	16.23 ± 0.14 a	40.27 ± 0.37 b	30.11 ± 0.30 de	11.11 ± 0.13 c
	4	15.10 ± 0.11 b	38.04 ± 0.27 c	29.59 ± 0.63 e	9.74 ± 0.34 d
	5	13.56 ± 0.11 d	37.26 ± 0.55 cd	30.93 ± 0.26 cd	8.67 ± 0.28 e
190	Control	12.91 ± 0.12 c	36.85 ± 0.77 b	34.79 ± 0.76 a	11.11 ± 0.19 b
	1	13.69 ± 0.28 b	40.23 ± 0.75 a	30.84 ± 0.47 b	11.85 ± 0.50 b
	2	14.69 ± 0.34 a	39.20 ± 0.38 a	30.22 ± 1.00 b	12.71 ± 0.76 a
	3	13.44 ± 0.48 b	36.94 ± 0.61 b	28.44 ± 0.86 c	10.11 ± 0.13 c
	4	12.83 ± 0.17 c	35.38 ± 0.83 c	28.25 ± 0.54 c	9.41 ± 0.45 c
	5	11.96 ± 0.12 d	35.26 ± 0.55 c	30.60 ± 1.27 b	8.00 ± 0.44 c

The data are given as the means of triplicate assays ± SD. The values were labeled with the letters a–f from the maximum to the minimum, such that values with different letters in the same column were significantly different (*p* < 0.05).

**Table 3:** Effect of superheated steam treatment on the foaming properties of wheat protein.

Steam Temperature (°C)	Treatment Time (min)	Wheat Flour		Gluten Protein	
		FA (%)	FS (%)	FA (%)	FS (%)
165	Control	8.0 ± 1.4 c	97.9 ± 1.0 a	8.0 ± 0.5 c	96.8 ± 0.3 a
	1	12.3 ± 1.1 a	97.2 ± 1.9 a	13.2 ± 0.6 ab	96.9 ± 0.3 a
	2	10.7 ± 0.6 ab	97.5 ± 0.4 a	14.2 ± 0.8 a	96.8 ± 0.1 a
	3	9.5 ± 1.0 bc	97.6 ± 0.9 a	13.0 ± 0.5 b	96.1 ± 0.3 b
	4	8.3 ± 1.1 c	97.4 ± 0.7 a	8.7 ± 0.8 c	95.4 ± 0.4 c
	5	4.2 ± 0.8 d	97.2 ± 0.5 a	8.3 ± 0.5 c	94.9 ± 0.3 c
190	Control	8.0 ± 1.4 c	97.9 ± 1.0 a	8.0 ± 0.5 d	96.8 ± 0.3 a
	1	19.3 ± 1.6 a	98.1 ± 0.3 a	16.8 ± 0.3 a	97.0 ± 0.2 a
	2	18.8 ± 1.8 a	96.9 ± 0.6 a	17.7 ± 0.3 a	96.8 ± 0.1 a
	3	14.3 ± 1.1 b	95.5 ± 0.2 b	14.7 ± 0.8 b	96.1 ± 0.3 b
	4	5.0 ± 0.7 d	97.7 ± 1.1 a	9.5 ± 0.9 c	94.7 ± 0.7 c
	5	4.0 ± 0.5 d	97.7 ± 0.3 a	7.0 ± 0.5 d	93.6 ± 0.3 d

FA and FS represent foaming ability and foam stability, respectively. The data are given as the means of triplicate assays ± SD. The values were labeled with the letters a–d from the maximum to the minimum, such that values with different letters in the same column were significantly different (*p* < 0.05).

**Table 4:** Effect of superheated steam treatment on the emulsifying properties of wheat protein.

Steam Temperature (°C)	Treatment Time (min)	Wheat Flour		Gluten Protein	
		EAI (m^2^/g)	ES (min)	EAI (m^2^/g)	ES (min)
165	Control	0.89 ± 0.02 f	27.1 ± 2.0 c	9.2 ± 0.3 b	28.1 ± 2.2 d
	1	1.19 ± 0.03 b	32.8 ± 0.4 b	12.2 ± 0.9 a	32.3 ± 2.5 c
	2	1.24 ± 0.02 a	34.8 ± 0.7 ab	12.4 ± 0.5 a	34.3 ± 1.4 abc
	3	1.14 ± 0.02 c	36.0 ± 1.0 a	10.9 ± 0.4 a	35.7 ± 0.4 ab
	4	1.09 ± 0.03 d	35.6 ± 0.3 a	11.8 ± 1.4 a	36.6 ± 0.3 a
	5	1.01 ± 0.03 e	32.5 ± 0.9 b	11.3 ± 0.5 a	32.9 ± 0.3 bc
190	Control	0.89 ± 0.02 d	27.1 ± 2.0 b	9.2 ± 0.3 c	28.1 ± 2.2 b
	1	1.07 ± 0.05 c	22.1 ± 0.2 c	10.7 ± 1.2 b	22.1 ± 0.4 c
	2	1.14 ± 0.02 b	21.8 ± 0.9 c	11.7 ± 0.4 ab	22.2 ± 0.3 c
	3	1.27 ± 0.03 a	26.6 ± 0.8 b	12.8 ± 0.6 a	25.6 ± 0.8 bc
	4	1.11 ± 0.04 bc	33.9 ± 2.5 a	11.0 ± 0.4 b	32.8 ± 3.4 a
	5	1.06 ± 0.02 c	35.2 ± 1.7 a	11.1 ± 0.4 b	36.2 ± 1.7 a

EAI and ES represent emulsifying activity index and emulsion stability, respectively. The data are given as the means of triplicate assays ± SD. The values were labeled with the letters a–d from the maximum to the minimum, such that values with different letters in the same column were significantly different (*p* < 0.05).

**Table 5:** Texture of cakes made from SS-treated soft wheat.

Steam Temperature (°C)	Treatment Time (min)	Hardness (g)	Springiness	Cohesiveness	Chewiness (N)	Resilience
165	Control	1566 ± 66 ab	0.762 ± 0.007 c	0.454 ± 0.009 b	541 ± 16 a	0.147 ± 0.004 c
	1	1393 ± 32 cd	0.815 ± 0.109 b	0.471 ± 0.009 a	533 ± 12 a	0.162 ± 0.006 b
	2	1327 ± 37 d	0.819 ± 0.014 b	0.477 ± 0.008 a	521 ± 7 a	0.171 ± 0.006 a
	3	1397 ± 20 cd	0.838 ± 0.005 a	0.436 ± 0.006 c	510 ± 4 a	0.162 ± 0.002 b
	4	1471 ± 67 bc	0.828 ± 0.005 ab	0.434 ± 0.004 c	535 ± 5 a	0.159 ± 0.003 b
	5	1593 ± 132 a	0.821 ± 0.012 b	0.415 ± 0.013 d	543 ± 45 a	0.156 ± 0.007 b
190	Control	1566 ± 66 a	0.762 ± 0.007 c	0.454 ± 0.009 b	541 ± 16 a	0.147 ± 0.004 d
	1	1301 ± 115 b	0.859 ± 0.011 a	0.495 ± 0.016 a	554 ± 60 a	0.178 ± 0.007 a
	2	1338 ± 16 b	0.858 ± 0.013 a	0.459 ± 0.004 b	554 ± 6 a	0.179 ± 0.007 a
	3	1343 ± 177 b	0.854 ± 0.012 ab	0.448 ± 0.021 bc	581 ± 37 a	0.179 ± 0.008 a
	4	1530 ± 51 a	0.848 ± 0.002 ab	0.431 ± 0.007 c	556 ± 5 a	0.166 ± 0.004 b
	5	1679 ± 85 a	0.838 ± 0.014 b	0.403 ± 0.020 d	568 ± 52 a	0.157 ± 0.007 c

The data are given as the means of triplicate assays ± SD. The values were labeled with the letters a–d from the maximum to the minimum, such that values with different letters in the same column were significantly different (*p* < 0.05).

## Data Availability

Data are contained within the article.
